# Spectrum of antibiotic resistance in UTI caused by *Escherichia coli* among HIV-infected patients in Uganda: a cross-sectional study

**DOI:** 10.1186/s12879-021-06865-3

**Published:** 2021-11-23

**Authors:** George Abongomera, Maurice Koller, Joseph Musaazi, Mohammed Lamorde, Marisa Kaelin, Hannington B. Tasimwa, Nadia Eberhard, Jan Hongler, Sabine Haller, Andrew Kambugu, Barbara Castelnuovo, Jan Fehr

**Affiliations:** 1 Department of Public and Global Health, Epidemiology, Biostatistics and Prevention Institute, University of Zurich, Hirschengraben 84, CH 8001 Zurich, Switzerland; 2 Makerere University Infectious Diseases Institute, Kampala, Uganda; 3 University Hospital Zurich, Infectious Diseases and Hospital Epidemiology, Zürich, Switzerland; 4 Epidemiology, Biostatistics and Prevention Institute, University of Zurich and University Hospital Zurich, Infectious Diseases and Hospital Epidemiology, Zürich, Switzerland

**Keywords:** Urinary tract infections, Antimicrobial resistance, HIV, Uganda, Sub-Saharan Africa

## Abstract

**Background:**

Antimicrobial drug resistance is one of the top ten threats to global health according to the World Health Organization. Urinary tract infections (UTIs) are among the most common bacterial infections and main reason for antibiotic prescription. The incidence of UTIs appears to be high among people living with HIV. We sought to determine the most common UTI pathogens among HIV infected patients and evaluate their susceptibility towards antibiotics.

**Methods:**

We performed a cross-sectional study among HIV-infected patients aged ≥ 18 years presenting at an HIV care specialized clinic with symptoms suggestive of a urethritis. Urine cultures were subjected to antibiotic susceptibility testing according to Clinical Laboratory Standards Institute. The data was analyzed using STATA, we performed Pearson’s Chi-square and Fisher’s exact tests to compare differences between proportions.

**Results:**

Out of the 200 patients, 123 (62%) were female. The median age was 41.9 years (IQR 34.7–49.3). Only 32 (16%) urine cultures showed bacterial growth. *Escherichia coli* was the most commonly isolated uropathogen (72%), followed by *Klebsiella pneumonia*e (9%). *E. coli* was completely resistant to cotrimoxazole and ampicillin; resistance to ciprofloxacin and ceftriaxone was 44% and 35% respectively; 9% to gentamicin; no resistance detected to nitrofurantoin and imipenem.

**Conclusions:**

Our findings are congruent with the Uganda national clinical guidelines which recommends nitrofurantoin as the first line antibiotic for uncomplicated UTI. Significant ciprofloxacin and ceftriaxone resistance was detected. In the era of emerging antibiotic resistance, understanding the local susceptibilities among sub-populations such as HIV infected patients is crucial. Further investigation is needed to address reasons for the low bacterial growth rate observed in the urine cultures.

**Supplementary Information:**

The online version contains supplementary material available at 10.1186/s12879-021-06865-3.

## Background

Urinary tract infections (UTIs) are one of the most common bacterial infections globally, with an estimated annual incidence of more than 150 million cases worldwide [1]. UTIs are one of the main reasons for the prescription of antibiotics [2, 3]. They are caused by a variety of uropathogens, most commonly gastrointestinal bacterial flora such as uropathogenic *Escherichia coli*, *Klebsiella pneumoniae*, various other *Enterobacteriaceae,* as well as *Staphylococcus saprophyticus* [4]. In recent years, studies have shown rising drug resistance patterns in these uropathogens across the globe [5, 6]. World Health Organization (WHO) surveillance data on antimicrobial resistance reveal high levels of resistance to a number of bacterial infections in both high and low-income countries [7]. Furthermore, antimicrobial resistance (AMR) was identified as one of the top ten threats to global health in 2019 that will require focus from health care providers and implementers [8].

Sub-Saharan Africa (SSA), a region with a high burden of infectious diseases, is especially vulnerable to antimicrobial resistance [9]. In South Africa, a study reported suboptimal adherence to national antibiotic prescription guidelines due to a number of factors [10]. These included undocumented diagnosis, unnecessary antibiotic prescription, incorrect dosage, incorrect drug and incorrect duration of therapy [10]. Studies conducted in Uganda revealed that up to 41% of antibiotics were issued over the counter and high antibiotic prescription rates among hospitalized patients with frequently missed doses [11, 12]. Antimicrobial resistance in UTIs to cotrimoxazole, ciprofloxacin, cephalosporins and other antibiotics have been reported in SSA, however the data is limited [13–15].

Generally, the incidence of UTIs appears to be high among people living with HIV, especially in those with lower CD4 lymphocyte count [16]. HIV-infected individuals with low CD4 lymphocyte count are often receiving prophylactic cotrimoxazole to prevent opportunistic infections, such as *pneumocystis jirovecii* pneumonia (PCP) [17]. Cotrimoxazole prophylaxis is not effective in reduction of the incidence of UTIs among HIV-infected individuals with lower CD4 lymphocyte count [16]. In Uganda, the national clinical guidelines for the treatment of UTI were revised, and cotrimoxazole was replaced with nitrofurantoin as a first-line agent for UTIs due to high resistance rates [18, 19].

This study was conducted through the Infectious Diseases Institute (IDI), Makerere University, Uganda and University of Zurich (UZH), Switzerland research collaboration. The study describes findings of a UTI surveillance project implemented in an out-patient HIV clinic of the IDI of the Makerere University College of Health Sciences in Kampala, Uganda. We sought to determine the most common UTI pathogens in the HIV-positive population and evaluate their susceptibility towards commonly prescribed antibiotic agents.

## Methods

### Study site

The study was conducted between September 2017 and December 2018 in an out-patient HIV clinic at IDI in Kampala, Uganda. It is an urban center of excellence for HIV care and treatment, with over 8000 patients.

### Study design and patients

We performed a cross-sectional study among HIV-infected patients aged ≥ 18 years presenting with symptoms suggestive of urethritis. This was defined as showing one or more of the following symptoms; dysuria, urgency, frequency and fever. The patient data was collected as part of routine surveillance at the out-patient HIV-Clinic for a period of 16 months.

### Urine sample collection

Patients were instructed on how to provide an adequate midstream urine sample (MSU) by a nurse with the help of a leaflet with visual step-by-step instructions of the procedure. The MSU was then taken to the laboratory at the Department of Medical Microbiology, Makerere University School of Biomedical Sciences within 2 h or within 24 h if refrigerated at 4 °C.

### Laboratory procedures

The MSU was subjected to urine dipstick and urine cultures were subjected to antibiotic susceptibility testing according to Clinical Laboratory Standards Institute (CLSI). Sheep Blood and Cystine Lactose Electrolyte Deficient (CLED) agar were used for urinary culture. Cultures were incubated at 35–37 °C and checked for growth after 18–24 h. Agar plates with pure growth > 10^4^ colony forming units (CFU)/ml, or > 10^5^ CFU/ml if two types of pathogens were present, were classified as showing significant growth and used for further identification and sensitivity testing using Müller-Hinton Agar. Plates with no visible colonies were reported as no bacterial growth. Growth results ≥ 10^2^ CFU/ml, but below the threshold for significant growth were reported as insignificant growth. Cultures with more than two types of organisms were reported as mixed growth, suggesting contamination. Drug susceptibility testing (DST) was performed for the following antimicrobial agents, as per the laboratory SOP: ciprofloxacin, cotrimoxazole, nitrofurantoin, ampicillin, ceftazidime, cefuroxime, ceftriaxone, chloramphenicol, gentamicin, imipenem, and nalidixic acid. Sensitivity was evaluated with the Kirby-Bauer disc diffusion method. More details on microbiology are provided in the Additional file 1: Appendix S1.

### Patient follow up

In case the cultured uropathogen showed resistance towards the empirically prescribed antibiotic regimen, patients were contacted and asked to report to the clinic. Therapy was then adjusted by the assigned follow-up physician according to the resistance test results.

### Data collection

Demographic and clinical characteristics, UTI related information including urine test results was collected using Epidata®. UTI-symptoms and further information about the patients’ medical history were obtained using a standard evaluation form that was filled out by the patients with the support of a nurse.

### Statistical analysis

The data was analyzed using STATA version 14.2 (College Station, Texas 77845 USA). Statistics for proportions, means with standard deviations (SD) and medians with interquartile ranges (IQR) were acquired to describe the data. We performed Pearson’s Chi-square and Fisher’s exact tests to compare differences between proportions.

## Results

A total of 200 HIV infected patients presenting with symptoms suggestive of UTI were recruited and included in the study. Of these, 123 (61.5%) were female. The median age was 41.9 years (IQR 34.7–49.3); 40.1 years (IQR 33.8–48.7) for females and 43.9 years (IQR 36.7–50.9) for males. The majority of patients presented with dysuria (158, 79.0%). Nearly all the patients (198, 99.0%) reported to be taking cotrimoxazole or dapsone prophylaxis, which was recommended by the Uganda HIV treatment guidelines at the time of the study. The median duration on cotrimoxazole or dapsone prophylaxis in months was 110 (IQR 64–142). 83 (41.2%) patients reported using other antibiotics within the past month, of these 59 (29.5%) were taking antibiotics at the time of recruitment. Almost all the patients, 195 (97.5%) were on antiretroviral treatment (ART) and the median duration on ART in months was 103 (IQR 60–144). Majority of them, 195 (97.5%) had had a viral load test within the past 12 months; of these 175 (87.5%) were undetectable (< 75 copies/ml). Comprehensive details on the baseline characteristics of patients recruited in the study are presented in Table 1.

**Table 1:** Characteristics of the HIV-infected patients who participated in the study

Characteristic	All participants n = 200	Female n = 123 (61.5)	Male n = 77 (38.5)
Demographic			
Median age (IQR)	41.9 (34.7–49.3)	40.1 (33.8–48.7)	43.9 (36.7–50.9)
UTI symptoms			
Dysuria	158 (79.0)	89 (72.4)	69 (89.6)
Urgency	116 (58.0)	75 (61.0)	41 (53.3)
Increased urine frequency	121 (60.5)	72 (58.5)	49 (63.6)
Fever (> 38.5 °C)	15 (7.5)	11 (8.9)	4 (5.2)
Comorbidities apart from HIV			
Diabetes mellitus	6 (3.0)	4 (3.3)	2 (2.6)
History of urological abnormalities	4 (2.0)	1 (0.8)	3 (3.9)
Others*	3 (1.5)	1 (0.5)	2 (2.6)
Antibiotics use			
Cotrimoxazole or Dapsone prophylaxis	198 (99.0)	122 (99.2)	76 (98.7)
Duration on Cotrimoxazole or Dapsonein months, median (IQR)	110 (64–142)	107 (64–143)	115 (61–142)
Recent use of other antibiotics^†^	83 (41.2)	54 (43.9)	29 (37.7)
Currently using other antibiotics	59 (29.5)	39 (31.7)	20 (26.0)
HIV history			
On ART regimen	195 (97.5)	121 (98.4)	74 (96.1)
Duration on ART in months, median (IQR)	103 (60–144)	99 (55–143)	111 (68–147)

Values are n (%) or IQR (25th, 75th percentile)

*CI* confidence intervals, *IQR* interquartile range, *UTI* urinary tract infection, *ART* antiretroviral therapy

*Chronic kidney impairment, neurogenic bladder, immunosuppression due to comorbidities.

^†^Within the last month

Out of 200 cultures, only 32 (16.0%) showed bacterial growth. 36 (18.0%) cultures showed mixed bacterial growth, and 132 (66.0%) cultures had no bacterial growth. *Escherichia coli* was the most commonly isolated uropathogen (71.9%), followed by *Klebsiella pneumoniae* (9.4%) and *Morganella morganii* (6.3%). Other isolates included *Acinetobacter baumannii, Enterobacter spp, Citrobacter freundii, Providencia spp., Enterococcus spp. and Group B Salmonella.*

*Escherichia coli* was completely resistance to ampicillin and cotrimoxazole. Resistance to ciprofloxacin and ceftriaxone was 44.4 and 35.0% respectively; to gentamicin was 9.1%, no resistance was observed to nitrofurantoin and imipenem. *Klebsiella pneumoniae* was cultured in three cases, all were resistant to cotrimoxazole and ampicillin. One out of two isolates was resistant to ciprofloxacin; one out of three to ceftriaxone, gentamicin and nitrofurantoin; none was resistant to imipenem. Table 2 shows a summary of the resistance profile of the isolated pathogens towards the tested antibiotics.

**Table 2:** Summary of resistance profiles of the isolated bacteria to antibiotics

Isolates	*Escherichia coli* n = 23		*Klebsiella pneumoniae *n = 3		*Morganella morganii *n = 2		Others^†^ n = 6	
Ciprofloxacin	8/18	(44.4)	1/2	(50)	1/2	(50)	3/6	(50)
Nalidixic acid	11/13	(84.6)	1/2	(50)	1/2	(50)	2/3	(66.6)
Ampicillin	13/13	(100)	2/2	(100)	1/1	(100)	3/5	(60)
Cotrimoxazole	20/20	(100)	3/3	(100)	2/2	(100)	4/5	(80)
Nitrofurantoin	0/20	(0)	1/3	(33.3)	1/2	(50)	2/4	(50)
Chloramphenicol	5/14	(36)	1/2	(50)	0/0	–	1/4	(25)
Ceftazidime	7/15	(46.7)	0/1	(0)	1/2	(50)	0/3	(0)
Cefuroxime	8/18	(44.4)	1/3	(33.3)	1/2	(50)	1/4	(25)
Ceftriaxone	7/20	(35)	1/3	(33.3)	1/2	(50)	0/3	(0)
Gentamicin	2/22	(8.1)	1/3	(33.3)	1/2	(50)	1/4	(25)
Imipenem	0/18	(0)	0/3	(0)	1/1	(100)	0/3	(0)

*Values are n (%)

^†^Others include: *Citrobacter freundii, Acinetobacter baumannii, Enterobacter spp., Group B Salmonella*

Table 3 compares culture growth, isolated pathogens and antibiotic resistance with and without recent use of other antibiotics in the past month (with exception of those on cotrimoxazole/dapsone prophylaxis). The antibiotics taken in the month prior to the study or being taken at the time of the study included doxycycline, metronidazole, amoxicillin, cefuroxime, ampicillin, azithromycin, gentamicin, ceftriaxone and chloramphenicol. No statistically significant difference was observed in cultural growth, pathogen distribution and antibiotic resistance between groups of the two groups of patients.

**Table 3:** Comparison of culture growth, isolated bacteria and resistance profile with and without previous antibiotic use (with exception of cotrimoxazole or dapsone prophylaxis)

	Overall	Previous antibiotic use		
	n = 200	Yes n = 83 (41.5)	No n = 117 (58.5)	p-values*
Culture growth				
Positive culture	32/200 (16)	12/83 (14.5)	20/117 (17.1)	0.62
Mixed culture	36/200 (18)	15/83 (18.1)	21/117 (18.0)	0.98
Insignificant bacterial growth	36/200 (18)	14/83 (16.9)	22/117 (18.8)	0.73
No bacterial growth	96/200 (48)	42/83 (50.6)	54 (46.2)	0.54
Isolated bacteria				
*Escherichia coli*	23/32 (71.9)	8/12 (66.7)	15/20 (75.0)	0.70
*Klebsiella pneumoniae*	3/32 (9.4)	1/12 (8.3)	2/20 (10)	0.99
*Morganella morganii*	2/32 (6.3)	1/12 (8.3)	1/20 (5)	0.99
Others†	6/32 (18.8)	3/12 (25)	3/20 (15)	0.65
Overall resistance profile				
Ciprofloxacin	11/26 (42.3)	6/11 (54.5)	5/15 (33.3)	0.23
Nitrofurantoin	3/28 (10.7)	2/9 (22.2)	1/19 (5.2)	0.09
Cotrimoxazole	28/29 (96.5)	11/12 (91.7)	17/17 (100)	0.41
Ceftriaxone	3/31 (9.7)	2/10 (20)	3/11 (27)	0.99

Values are n (%)

*Fisher’s Exact test

^†^Others include: *Acinetobacter baumannii, Citrobacter freundii, Enterobacter spp., Group B Salmonella,*

*Providencia spp., Enterococcus spp*

## Discussion

This surveillance study describes urinary tract pathogens and their resistance patterns among Ugandan patients living with HIV who presented with symptoms suggestive of a UTI. The most commonly isolated pathogen in our study was *Escherichia coli* (71.8%). This finding is congruent with other studies conducted in Uganda, Sub-Saharan African countries and other parts of the world. In Uganda, two studies found *Escherichia coli* to be the responsible pathogen in 57.5% and 50% of culturally confirmed UTI respectively [14, 20]. A study in Ethiopia, reported *Escherichia coli* in 52.7% of urine cultures [21]. In Europe and North America, *Escherichia coli* is estimated to be responsible for 60–70% of UTIs [22, 23]. However, there is limited antimicrobial surveillance data for UTI among HIV infected patients in Uganda. Hence a comparison of the resistance profiles of pathogens with other studies conducted locally was not possible.

*E. coli,* the most isolated uropathogen was completely resistant to ampicillin and cotrimoxazole, as well as significantly resistant to ciprofloxacin and ceftriaxone. Susceptibility was high to nitrofurantoin, imipenem and gentamicin. In Uganda, high resistance in uropathogens to cotrimoxazole, amoxicillin, and nalidixic acid and considerable resistance towards ciprofloxacin has been observed [17]. This resistance pattern is similar to findings in other SSA countries [18]. Another Ugandan study describes high sensitivity towards nitrofurantoin, with similarly low sensitivity towards ciprofloxacin and cotrimoxazole [14]. High resistance towards cotrimoxazole has been described in other studies conducted in Sub-Saharan African countries and could be attributed to the use of cotrimoxazole as prophylaxis against opportunistic infections in HIV-positive patients [14, 17, 24]. It is noteworthy that ampicillin and other beta-lactam antibiotics are widely dispensed over the counter in Uganda without the need of a physician’s prescription [11]. This fact could provide an alternative explanation to the high resistance rates observed. In other patient scenarios, the recommended duration of the antibiotic treatment is cut short due to financial constraints [11, 25]. A study in Uganda reported ciprofloxacin and ceftriaxone as commonly prescribed systemic antibiotics in hospitalized patients [12]. The same study reported that under-administration of prescribed antibiotics was frequent and therefore a concern for loss of efficacy and emergence of antibiotic resistance. In the current Ugandan clinical guidelines, ciprofloxacin is recommended as second line therapy in UTIs [22]. However, this study has demonstrated that ciprofloxacin has been ineffective in inhibiting bacterial growth in vitro. High efficacy of nitrofurantoin and gentamicin has been described in Sub-Saharan African countries [14, 18, 20]. Nitrofurantoin is recommended as the first line agent for uncomplicated UTIs by the Ugandan clinical guidelines and the study results concur with this recommendation [22]. Since gentamicin and imipenem are administered intravenously and are considered as antibiotics reserved for difficult to treat situations, this may limit their dispensation over the counter and overall availability and may provide an explanation of their low resistance rates.

Out of 200 patients, only 32 (16%) cultures had significant bacterial growth. Other studies in Sub-Saharan African countries showed similarly low rates of culture growth [14, 17, 20, 26]. In comparison, studies conducted in other settings such as Europe showed high growth rates of around 80% [27, 28]. Possible explanations for the low growth rate might be found in the high percentage of patients with recent antibiotic consumption (41.5%) and the use of cotrimoxazole or dapsone prophylaxis in almost all patients (99.0%). The consumed antibiotic agents may inhibit in vitro culture growth of bacteria in urine. Other studies have shown such an inhibiting impact of antibiotic intake on urine culture growth [29]. As both groups in this analysis were taking cotrimoxazole/dapsone prophylaxis, additional antibiotic intake may not significantly further alter positive culture growth. Another potential reason for the low rate of bacterial growth on culture might be that some of the patients’ symptoms were not attributed to a UTI. Alternative causes of the described symptoms may be found in sexually transmitted diseases (STI) such as chlamydia or gonococcal urethritis which cannot be diagnosed by standard urine cultures.

The limitations of this study are we included only HIV-infected patients presenting with symptoms suggestive of urethritis. Furthermore, we did not have recent CD4 count results of the patients because CD4 is no longer routinely measured and replaced by viral load count for monitoring purposes of HIV patients. Lastly, we did not collect data on previous hospitalizations of the patients.

## Conclusion

Our study found the most isolated pathogen, *E. Coli* to be completely resistant to cotrimoxazole and ampicillin. Furthermore, significant resistance to ciprofloxacin and ceftriaxone was observed. Susceptibility was high to gentamicin, nitrofurantoin and imipenem. It is noteworthy that majority of urine cultures showed no bacterial growth in symptomatic patients. This is a concern and further studies are needed to address potential reasons behind the observed low growth rate. Finally, in the era of emerging antibiotic resistance, understanding the epidemiology and the local susceptibilities among sub-populations such as HIV infected individuals is crucial. This study is aligned with the WHO global action plan on antimicrobial resistance and provides further information that will contribute to the database on antimicrobial resistance in UTI in Uganda.

## Supplementary Information

**Additional file 1: Appendix S1.** SOP Microbiology.

## Data Availability

All data generated during this study are included in this article. Details of the full data may be accessed through the Corresponding Author, George Abongomera, Department of Public and Global Health, Epidemiology, Biostatistics and Prevention Institute, University of Zurich, Hirschengraben 84, CH 8001 Zurich, Switzerland, Phone: + 41 44 634 49 36, Email: george.abongomera@uzh.ch.

## Abbreviations

AMR: Antimicrobial resistance
ART: Antiretroviral therapy
CLED: Cystine Lactose Electrolyte Deficient
CLSI: Clinical Laboratory Standards Institute
DST: Drug susceptibility testing
IDI: Infectious Diseases Institute
IQR: Interquartile range
MSU: Midstream urine
PCP: *Pneumocystis jirovecii* Pneumonia
SD: Standard deviations
SSA: Sub-Saharan Africa
UTIs: Urinary tract infections
UZH: University of Zurich
WHO: World Health Organization
