# Supplementary material for: Spectrum of antibiotic resistance in UTI caused by Escherichia coli among HIV-infected patients in Uganda: a cross-sectional study
Source: BMC Infect Dis. 2021 Nov 23;21:1179. doi: 10.1186/s12879-021-06865-3 (PMC8609806; doi:10.1186/s12879-021-06865-3)
Supplement: Supplementary file 1 — Additional file 1: Appendix S1. SOP Microbiology. [file 12879_2021_6865_MOESM1_ESM.pdf]

Appendix 1: SOP Microbiology

MAKERERE UNIVERSITY

Scope and application

Urine is examined for diagnosis of urinary tract infections including: cystitis, pyelonephritis, asymptomatic bacteriuria and less commonly acute prostatitis, pyelonephric abscess and urosepsis.

Urine specimens can be divided into 3 categories including:

- Voided urine: Midstream urine, neonatal bagged urine, indwelling (Foley catheter) urine, ileal conduit urine and suprapubic catheter urine

- In and out catheter urine

- Aseptically collected urine: Suprapubic aspirate urine, Nephrostomy urine

Determining of significance of organisms isolated from urine that is not aseptically collected depends on quantitative culture with defined thresholds for significant growth. These thresholds differ depending on the method of collection.

1.1. Objectives:

1.1.1. To isolate, identify bacteria responsible for UTI and determine their antimicrobial susceptibility profiles

1.1.2. To detect leukocytes, yeasts, casts, squamous epithelial cells and parasites on microscopy

1.1.3. To perform glucose, protein and other biochemical assays as requested

2. Responsibilities:

2.1. Reception, cataloguing and processing of the specimen will be done by a technician Reception, cataloguing, processing of the specimen and reporting of results shall be done by a trained laboratory assistant/technician.

2.2. The overall process shall be under the supervision of a microbiologist

3. Acceptable specimens:

The following specimens if properly labeled and received within 24 hours of collection will be accepted:

3.1. Midstream urine
3.2. In dwelling catheter urine (collected by aseptic puncture of the catheter tubing)
3.3. In and out catheter urine
3.4. Suprapubic aspirated urine
3.5. Cystoscopy specimens

4. Rejection of specimens:

The following specimens will be rejected:

4.1. Bedpan urine

4.2. Condom/Texas catheter urine
4.3. Specimens received in non-sterile containers
4.4. Bag urine
4.5. Specimens that have leaked out of the container
4.6. Urinary catheter tips
Always inform the ward/clinic when rejecting the specimen

5. Safety issues:
Use personal protectives including gloves and observe standard precautions.

6. Equipment and materials:
6.1. Equipment
- 6.1.1. Incubator at 35 – 37°C
- 6.1.2. Microscope
- 6.1.3. Candle jar/carbon dioxide incubator
- 6.1.4. Inoculating loops (10µl)
- 6.1.5. Plastic centrifuge tubes (15ml)

6.2. Culture media
- 6.2.1. Sheep blood agar
- 6.2.2. CLED agar
- 6.2.3. Sabouraud dextrose agar

6.3. Reagents/supplies
- 6.3.1. Urine examination strips
- 6.3.2. Microscope slides

7. Procedure:
7.1. Macroscopic appearance:
Examine for and record:
- 7.1.1. Color: e.g. colorless, pale yellow, deep yellow,
- 7.1.2. Consistence: e.g. Clear, turbid

7.2. Perform biochemical tests using dipsticks:
- 7.2.1. Protein: done routinely (on all specimens)
- 7.2.2. Glucose: done routinely (on all specimens)
- 7.2.3. Other tests e.g. ketones are done on request

Record results as Nil, 1+, 2+ etc. or quantity of the parameter tested according to the color changes as indicated on the strip container.

7.3. Microscopic examination:
- 7.3.1. Centrifuge a portion of the urine at 2000RPM for 5 minutes
- 7.3.2. Examine the sediment under high power (x40 objective) for: leukocytes, red cells, epithelial cells, casts, crystals, bacteria, yeast cells and parasites.
- 7.3.3. Quantify the red cells, epithelial cells, casts, crystals, bacteria, yeast cells and parasites as: **occasional, +, ++, +++** depending on the number seen.
- 7.3.4. Quantify the number of leukocytes (pus cells) as the number seen per high power field as <5 WBC/HPF, 6-10 WBC/HPF, 21 - 40 WBC/HPF or full field

7.4. Culture
Day one (Inoculation of the plates)

7.4.1. Mix the urine well

7.4.2. Hold a calibrated wire loop (10µl) vertically and immerse it just below the surface of the urine

7.4.3. Streak the loop down the center of a plate of blood agar and without flaming, use the same loop to make cross-streaks at a 90° angle to the original streak. These should be close together and should extend toward the edge of the plate

7.4.4. Repeat steps b to d for a CLED plate

7.4.5. Incubate the plates aerobically at 35-37°C

Day 2 (Reading the cultures)

7.4.6. Inspect plates for growth and determine colony count (refer to table 1 below)

7.4.7. Perform identification and antimicrobial susceptibility tests on potential pathogens present in significant numbers (refer to table 1 below)

Table 1: Quantification of colonies (Using 10µl loop)

Number of colonies on plate	CFU/ml
1-9	10^2
10-99	10^3
100	10^4
>100	$>10^4$

Table 2: Interpretation of Colony Counts for Urine

Detected growth	Midstream urine	In out Catheter urine	Suprapubic/ cystoscopy urine
1 type of organism	< 10^4CFU/ml<br>(< 100 Colonies on plate)<br>Growth not significant[1]<br><br>≥ 10^4CFU/ml<br>(≥100 Colonies on plate)<br>Growth is significant<br>Do identification and sensitivity	≥ 10^4CFU/ml<br>(≥100 Colonies on plate)<br>Growth is significant<br>Do identification and sensitivity	Any growth is significant do identification and sensitivity
2 types of organisms	Each ≥ 10^5CFU/ml<br>2 probable pathogens in equal amounts<br>Do identification and sensitivity on both	Each ≥ 10^4CFU/ml<br>2 probable pathogens in equal amounts<br>Do identification and sensitivity on both	Any growth is significant do identification and sensitivity
2 types of organisms with one predominant	1st org > 10^5CFU/ml<br>2nd org < 10^5CFU/ml<br>Do Identification and sensitivity on dominant organism only	1st org > 10^4CFU/ml<br>2nd org < 10^4CFU/ml<br>Do Identification and sensitivity on dominant organism only	Any growth is significant do identification and sensitivity
> 2 types of organisms	Mixed growth probably due to contamination, request a repeat	If one isolate is dominant (>10^4 CFU) it is considered significant, do ID and Sensitivity.<br>Consider isolates < 10^4CFU/ml as contaminants, request a repeat	Any growth is significant do identification and sensitivity
[1]Growth may be significant in symptomatic males, females with acute dysuria, patients on antibiotics, patients with chronic or recurring infections, children < 12 years of age<br><br>**Note: Consider any growth of diphtheroids, Lactobacillus, viridans streptococci and bacillus spp as not significant despite the degree of growth**			

8. Reporting of Results:

8.1. Report:

8.1.1. Macroscopic appearance: Color and consistence

8.1.2. Chemistry findings: Amount of protein and glucose (and other parameters if requested)

8.1.3. Microscopic findings: Leukocytes per high power field

8.1.4. Culture findings the number of different types of bacteria and their significance. (Refer to table 2)

8.1.5. For significant growth, report the antimicrobial susceptibility profiles

References:

1. Toronto Medical Laboratories / Mount Sinai Hospital Microbiology Department. Policy # MI\UR\01\v04. Urine culture manual

2. Microbiology QA practices and procedures, College of Physicians and Surgeons of Saskatchewan, 2004

3. National Health Services and Health Protection Agency National Standards Methods: BSOP 41. Investigation of Urine.

4. Cheesbrough, M. (1985). Medical Laboratory Manual for Tropical Countries. Oxford, ELBS

Appendix 2: Protocol summary

Safety issues

- Follow standard precautions

Materials required

- Microscope slides
- Blood agar plate
- CLED agar plate
- Sabouraud dextrose agar plate
- Urine examination strips
- 1 or 10µl inoculating loops

Specimens/collection

- Voided urine:
  - Mid stream
  - In dwelling catheter (Collected by aseptic puncture of catheter tubing)
- In and out catheter urine:
  Urine collected upon 'new' catheterization.
- Aseptically collected urine:
  e.g. supra-pubic aspirate, nephrostomy urine

ACCESSION SPECIMEN INTO DATA BASE

Note macroscopic appearance:

- Colour and consistency

Microscopy:

- Centrifuge a portion at 2000RPM for 5 min and examine sediment at x40 for WBC, RBC, Epith cells, casts, yeasts, parasites
- May be omitted for antenatal clinic screening for asymptomatic bacteriuria

Chemistry:

- Detect presence of glucose, proteins, ketones using dip sticks

Culturing

- Inoculate plates of CLED/MacConkey and blood agar with 10µl of well mixed urine and streak for discrete colonies
- Incubate aerobically at 35°C for 18 – 24 hours

Plate reading (18 – 24 hrs):

- Quantify each colonial types
  - 1-9 colonies = 10^2 CFU/ml
  - 10 – 99 col = 10^3 CFU/ml
  - 100 colonies = 10^4 CFU/ml
  - 1000 colonies = 10^5CFU/ml

Follow up of voided urine cultures:

No of isolates	Colony counts/ml	Workup
Any	$<10^4$	Ignore
1	10^4	ID & Sense
2	Both > 10^5	ID & sense both
2	One > 10^5 --<br>One < 10^5 --	----ID & sense<br>----Ignore
2	Both < 10^5 -	---- No workup
≥ 3	Any quantity	-----No workup

ID = Identification

**** Ignore any number of Lactobacillus, viridan streptococci, diphtheroids, Bacillus spp**

Counts of 10^2/ml could be significant for patients with symptomatic UTI.

Follow up for in/out catheter urine

No of isolates	Colony counts/ml	Workup
Any	$<10^4$	Ignore
1	10^4	ID & Sense
2	Both > 10^4	ID & sense both
2	One > 10^4 --<br>One < 10^4 --	----ID & sense<br>---- Describe (Do not follow up)
2	Both < 10^4 -	---- No workup
≥ 3	> 10^4<br>Any < 10^4	-----ID & Sense<br>---- Ignore

Counts of 10^2/ml could be significant for patients with symptomatic UTI.

**** Ignore any number of Lactobacillus, viridan streptococci, diphtheroids, *Bacillus spp***

Follow up of aseptically collected urine:

Identify and perform sensitivity for any number/type of organisms isolated

Reporting

- Macroscopic appearance
- Chemistry findings
- Microscopic findings
- Culture findings:-
  - No bacterial growth
  - No significant growth
  - Significant growth with ID & sensitivity

Turn around time

- 24 hours if no significant growth
- 48 - 72 hours for significant cultures
